# Cluster segmentation and stereo vision-based apple localization algorithm for robotic harvesting

**DOI:** 10.3389/fpls.2025.1598414

**Published:** 2025-11-27

**Authors:** Jianxia Wang, Wenbing Sun

**Affiliations:** College of Cyber Security, Tarim University, Alar, China

**Keywords:** apple detection, stereo vision system, orchard robotics/robotic harvesting, clustering-based segmentation, 3D localization, precision agriculture

## Abstract

**Introduction:**

Automated apple harvesting is hindered by clustered fruits, varying illumination, and inconsistent depth perception in complex orchard environments. While deep learning models such as Faster R-CNN and YOLO provide accurate 2D detection, they require large annotated datasets and high computational resources, and often lack the precise 3D localisation required for robotic picking.

**Methods:**

This study proposes an enhanced K-Means clustering segmentation algorithm integrated with a stereo-vision system for accurate 3D apple localisation. Multi-feature fusion combining colour, morphology, and texture descriptors was applied to improve segmentation robustness. A block-matching stereo model was used to compute disparity and derive 3D coordinates. The method was evaluated against Faster R-CNN, YOLOv7, Mask R-CNN, SSD, DBSCAN, MISA, and HCA using metrics including Recognition Accuracy (RA), mean Average Precision (mAP), Mean Coordinate Deviation (MCD), Correct Recognition Rate (CRR), Frames Per Second (FPS), and depth-localisation error.

**Results:**

The proposed method achieved >91% detection accuracy and <1% localisation error across challenging orchard conditions. Compared with Faster R-CNN, it maintained higher RA and lower MCD under high fruit overlap and variable lighting. Depth estimation achieved errors between 0.4%–0.97% at 800–1100 mm distances, confirming high spatial accuracy. The proposed model exceeded YOLOv7, SSD, FCN, and Mask R-CNN in F1-score, mAP, and FPS during complex lighting, occlusion, wind disturbance, and dense fruit distributions.

**Discussion and Conclusion:**

The clustering-based stereo-vision framework provides stable 3D localisation and robust segmentation without large training datasets or high-performance hardware. Its low computational demand and strong performance under diverse orchard conditions make it suitable for real-time robotic harvesting. Future work will focus on large-scale orchard deployment, parallel optimisation, and adaptation to additional fruit types.

## 1 Introduction

The apple is one of the most popular fruit crops, ranking second in global fruit production. Harvesting apples remains a crucial yet demanding operation because it requires substantial labor and time (Qu et al., 2015; Jia et al., 2020). Traditional harvesting methods rely primarily on manual workforces, resulting in increased expenses, workforce shortages, and inconsistent quality and efficiency. Researchers have extensively investigated automated fruit detection and harvesting technologies that utilize machine vision and clustering-based segmentation to boost efficiency and precision (Tu et al., 2010; Jia et al., 2020).

In recent years, deep learning techniques such as YOLO, SSD, Faster R-CNN, and Mask R-CNN have been widely applied in fruit detection and recognition (Onishi et al., 2019; Biffi et al., 2020; Jia et al., 2020; Zhang et al., 2020; Xiao et al., 2023, 2023). These systems fall into two categories: single-stage models (e.g., YOLO, SSD), which directly predict object locations and classes for faster processing, and two-stage models (e.g., Faster R-CNN, Mask R-CNN), which first propose candidate regions to improve classification and bounding accuracy (Tianjing and Mhamed, 2024; Shi et al., 2025) (Likas et al., 2003; Wang et al., 2022; Mhamed et al., 2024; Tianjing and Mhamed, 2024). Recent studies have demonstrated the potential of UAV-based phenotyping and machine learning approaches for monitoring crop traits and yield in tomato and quinoa, highlighting the growing role of computer vision in precision agriculture (Johansen et al., 2019, 2020; Jiang et al., 2022a). Deep learning enhances fruit detection by extracting key colour, shape, and texture features for segmentation and recognition. However, accuracy in orchards is hindered by variable lighting, foliage cover, and clustered fruit. Moreover, reliance on large datasets, high computational demands, and long training times limits their practical use in apple harvesting (Wang et al., 2022). Moreover, they often produce only 2D bounding boxes, lacking the precise in-depth information needed for robotic harvesting. These constraints limit their suitability for real-time field deployment.

Beyond fruit detection, deep learning has advanced applications in remote sensing, radar imaging, and ecological monitoring (Guan et al., 2025). Recent studies on PolSAR ship detection (Gao et al., 2023a), scattering-aware networks, few-shot SAR classification (Gao et al., 2023b, 2024), and multi-source data fusion highlights its versatility in complex detection tasks (Shen et al., 2024; Zhang et al., 2024). These cross-domain advances reinforce the relevance of developing efficient and adaptable methods for automated fruit detection and localization.

An alternative to deep learning is clustering-based segmentation. K-Means clustering is an unsupervised learning method that groups pixels by feature similarity, enabling effective fruit segmentation under complex orchard conditions ( (Likas et al., 2003; Na et al., 2010). K-Means delivers rapid and sturdy segmentation, which stands out from other methods like Fuzzy C-Means and DBSCAN, which require more computation and struggle with noise (Song et al., 2013; Jamel and Akay, 2019; Ikotun et al., 2023). Previous studies have applied K-Means for apple recognition (Wang Dandan et al., 2015). While some researchers utilized integrated extremum methods for fruit positioning (Jia et al., 2020). Recent studies further refined segmentation with fuzzy C-means (Sarbaini et al., 2022) CNN-based semantic segmentation (Ramadhani et al., 2022; Wang et al., 2022), and monocular vision approaches (Zubair et al., 2024). However, the challenge of achieving robust performance in real orchard conditions with limited data remains (Yang et al., 2012).

This study presents an enhanced K-Means clustering segmentation algorithm combined with multi-feature fusion (colour, morphology, and texture) and stereo vision for accurate 3D localization. The approach is designed to reduce misclassification and provide depth information critical for robotic harvesting. Unlike deep learning methods, the proposed system emphasizes computational efficiency, real-time applicability, and reduced training data requirements, making it well suited to practical orchard deployment. The method is comprehensively evaluated against state-of-the-art models, including Faster R-CNN, YOLOv7, and Mask R-CNN, and demonstrates superior accuracy, reduced coordinate deviation, and stable performance across different camera angles.

## 2 Materials and methods

The experimental setup consists of a four-arm parallel picking robot equipped with a high-precision vision system and a 3D stereo camera (1920 × 1080 pixels; Model: Hikvision MV-DL2125-04H-R) for apple detection and localization. The 3D camera was mounted at the front end of the robotic arm. Computational processing was performed on a high-performance computer running an Intel i7–12700 processor, ensuring efficient execution of clustering, segmentation, and localization tasks. Apple images were collected from a commercial orchard with diverse lighting conditions (morning, noon, evening), varying shading levels, and different apple clustering patterns to ensure a representative dataset. A dataset comprising 4,200 sample images of Aksu apples, a variety cultivated in Aksu Prefecture, Xinjiang, China, was collected. The dataset includes 2,200 images of red apples against green foliage and 2,000 images of green apples against green foliage. Each apple within the images was manually annotated using a circle-fitting method to ensure precise localization and segmentation. The dataset was split into an 8:2 ratio, with 80% used for training and 20% for testing. This choice ensured sufficient data for training while maintaining an independent set for performance evaluation. As the proposed method is based on clustering and does not require iterative hyperparameter optimization, no separate validation set was used. A similar adjustment of dataset splitting has been discussed in previous studies with small datasets (Ashtiani et al., 2021). Each image was manually annotated using LabelImg software, and apples were labelled based on their position, size, and occlusion level. To improve the model’s robustness, data augmentation was applied. Random rotation (± 15°), brightness variation (± 20%), and Gaussian noise were introduced to simulate real-world orchard variability caused by lighting changes, fruit occlusion, and viewing angle differences. This process reduced the risk of overfitting and enabled better generalization to unseen samples. Similar to findings in postharvest imaging studies (Javanmardi and Ashtiani, 2025), such augmentation strategies enhance dataset diversity and improve the reliability of classification models.

In the next section, Equations describe standard image preprocessing operations, clustering formulations, stereo vision disparity and depth estimation, and evaluation metrics are based on established methods documented in (Hartigan and Wong, 1979; Hartley and Zisserman, 2003; Gonzales and Woods, 2018). The enhanced K-means clustering and stereo vision localization method was implemented using standard Python and OpenCV libraries, with all parameters reported in this study. The dataset cannot be made publicly available due to restrictions, but a representative subset or implementation details are available from the corresponding author upon reasonable request.

### 2.1 Optimization of apple image segmentation using enhanced K-Means

Combining morphological processing, feature optimization, and colour space analysis, a modified K-Means clustering method was constructed. Enhanced colour sensitivity was achieved by converting RGB to HSI, using the H component for exceptional target-background difference. Images were filtered using Gaussian and median filtering techniques to reduce noise (**Supplementary Equation 2**) and then transformed to greyscale to ensure consistency under varying illumination conditions (**Supplementary Equation 1**).

**Figure 1:**
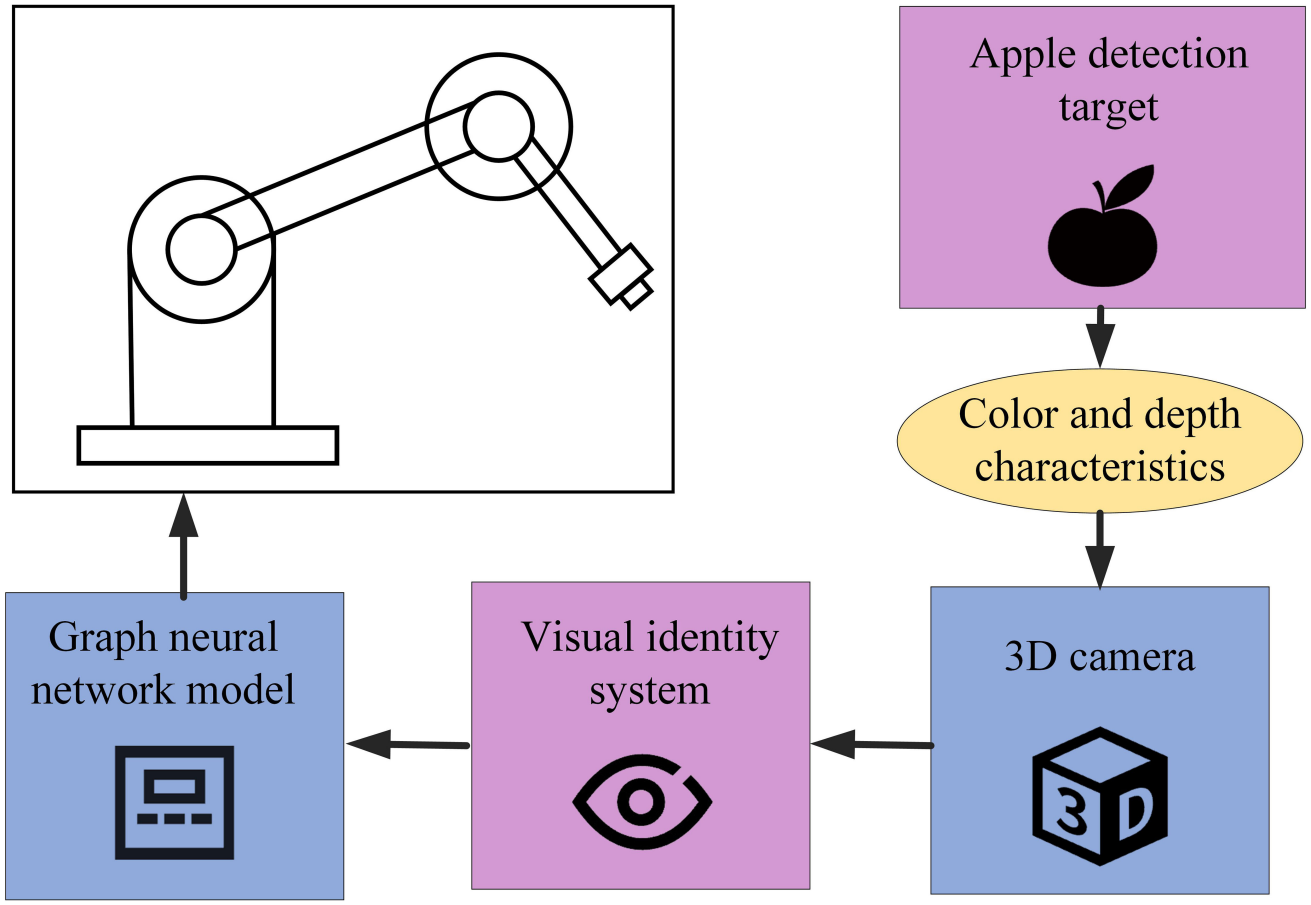
Conversion method from RGB to HSI color space.

Then, we extracted the HSI colour space that is highly sensitive to apple colour for segmentation purposes using Equation 1. The RGB colour space illustrated variations in colour intensity and brightness, whereas the HSI space replicated human visual perception abilities. As **Figure 1** shows, the RGB to HSI conversion turned unit square data into a bicone. A 3D camera captured apple image features and stored them as RGB grayscale values, ensuring enhanced consistency for segmentation under variable lighting conditions.

(1) $$H=\arctan\left(\frac{\sqrt{3}(G-B)}{2R-G-B}\right)$$

Where *H* indicates component values.

The H component proved useful for separating apples from the background. The conventional K-Means method did, however, show errors, including mis-segmentation in challenging environments. To improve accuracy and robustness, the algorithm was enhanced through an adaptive selection of the initial clustering centers (Equations 2, 3). The updated clustering method minimized intra-cluster variance (Equation 5).

(2) $$C_{k}=argmax_{P(i)}\sum_{j\in N(i)}\frac{1}{∥H(i)-H(j)∥}$$

Where *C_k_* denotes the initial center of the k class; *P_(i)_*denotes the set of points; *N _(i)_* denotes the set of domain points; *H* (*i*) and *H* (*j*) represent the feature vectors or attribute values of pixels *i* and *j*.

(3) $$D(x^{0},y^{0})=\sqrt{\sum_{m=1}^{n}w_{m}\cdot {\left(F_{m}(x^{0})-F_{m}(y^{0})\right)}^{2}}$$

Where *D* (*x*^0^, *y*^0^) is the Euclidean distance between the pixel point *x*^0^ and *y*^0^ and wm for the feature weights; *n* denotes the total dimension of the feature space; *F_m_* (*x*^0^) and *F_m_* (*y*^0^) represent the pixel intensities in pixels *x*^0^ and *y*^0^ in the m^th^ dimension, respectively.

**Figure 2:**
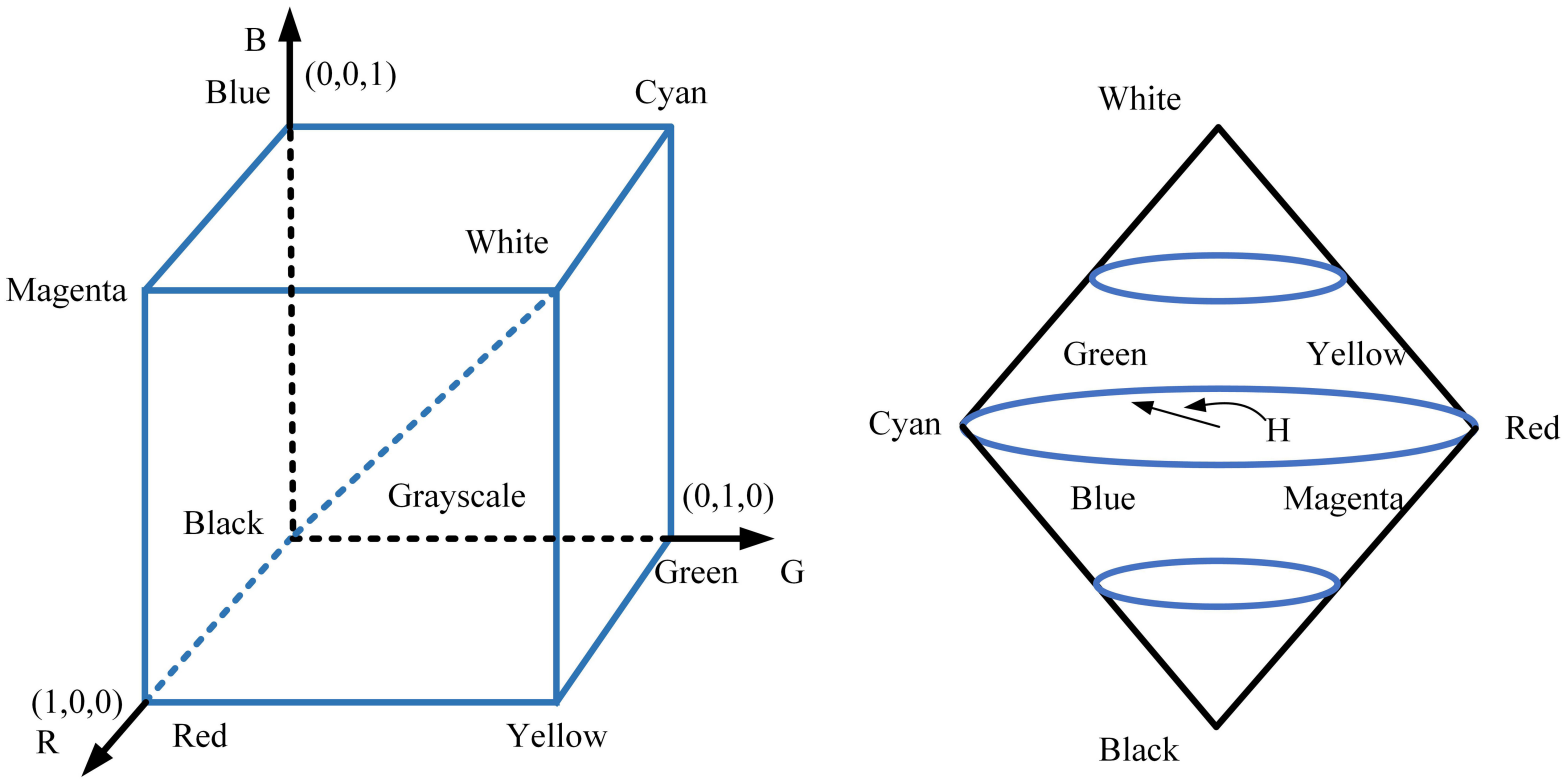
Morphological boundary extraction through erosion and subtraction. Small artifacts are removed, and clean object edges are restored for clustering.

The segmentation results underwent morphological processing, eliminating small noise elements and restoring target edges (**Supplementary Equation 3**). Boundary extraction utilized erosion to isolate object edges, as shown in **Figure 2**. Connected region calculation was performed using **Supplementary Equation 4** to obtain complete target information.

### 2.2 Multi-feature model for apple recognition and 3D positioning

**Figure 3:**
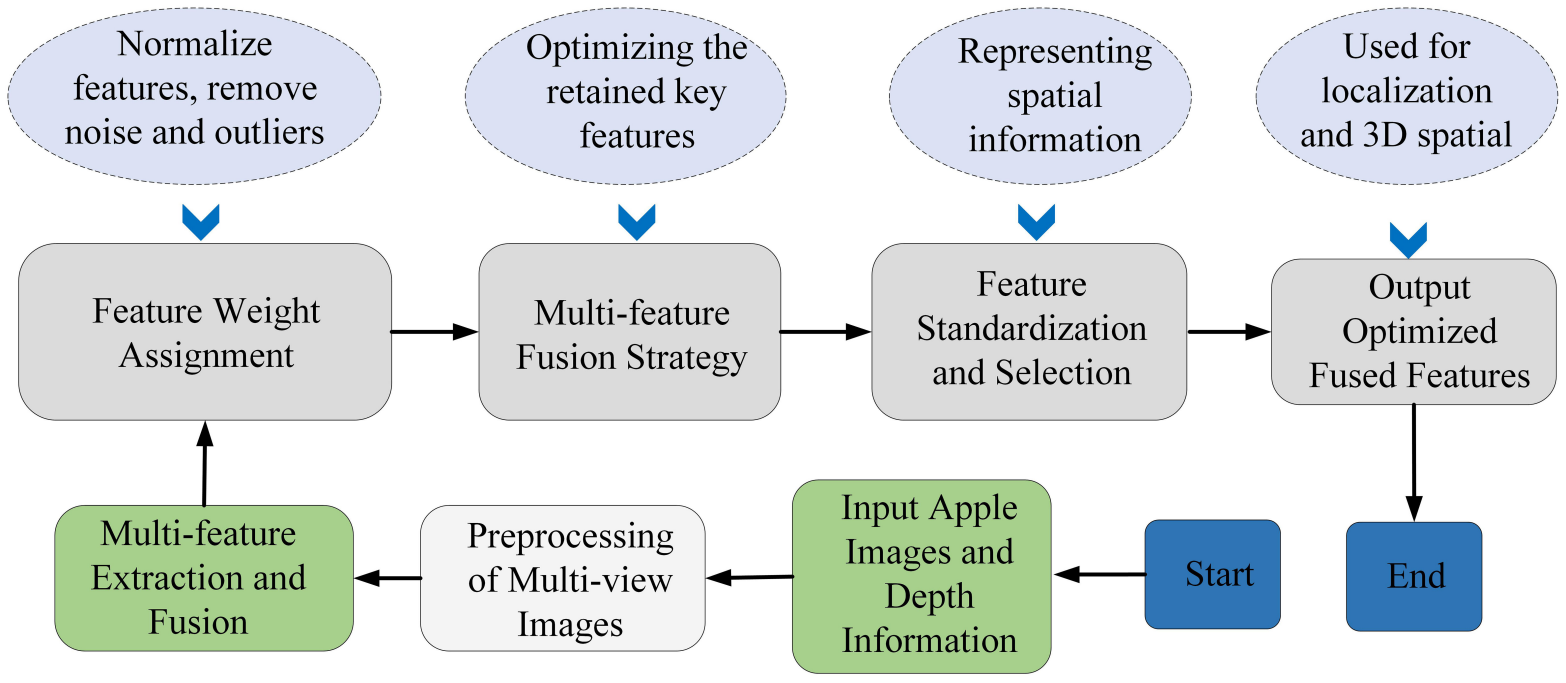
Schematic of the robotic apple detection system integrating a 3D camera, a visual identity module, and a graph neural network for precise recognition.

Following segmentation and clustering, apple centroids were precisely recognized by integrating colour, morphology, and texture features. Stereo vision technology and 3D camera calibration principles were used to map apples from 2D image coordinates to 3D spatial coordinates, providing accurate positional data for the harvesting robot. **Figure 3** displays the calibration principle for the stereo vision system and the 3D camera. The stereo vision system and 3D camera underwent calibration to synchronize the vision coordinate system with the robot coordinate system, which enabled precise target recognition and localization.

Single-feature detection showed high vulnerability to environmental conditions, including lighting and noise levels. Therefore, a multi-feature fusion approach was employed to enhance detection robustness and accuracy. Composite feature values determined target areas based on colour, texture, and morphology weights (Equation 4).

(4) $$T(x,y)=\alpha_{1}H(x,y)+\alpha_{2}\text{GLCM}(x,y)+\alpha_{3}\text{Shape}(x,y)$$

Where *T* (*x*, *y*) is the composite feature value, which is used to determine whether the pixel point belongs to the target area or not; *α*_1_, *α*_2_ and *α*_3_ are the weight coefficients, corresponding to the weights of colour, texture and morphological features, respectively. The values of α₁, α₂, and α₃ were empirically tuned using the training dataset, selecting the combination that achieved the best segmentation and detection performance under varying orchard conditions. *H* (*x*, *y*) indicates a colour feature; GLCM (*x*, *y*) denotes the grayscale covariance matrix, which is used to extract texture features; Shape (*x*, *y*) represents morphological features.

**Figure 4:**
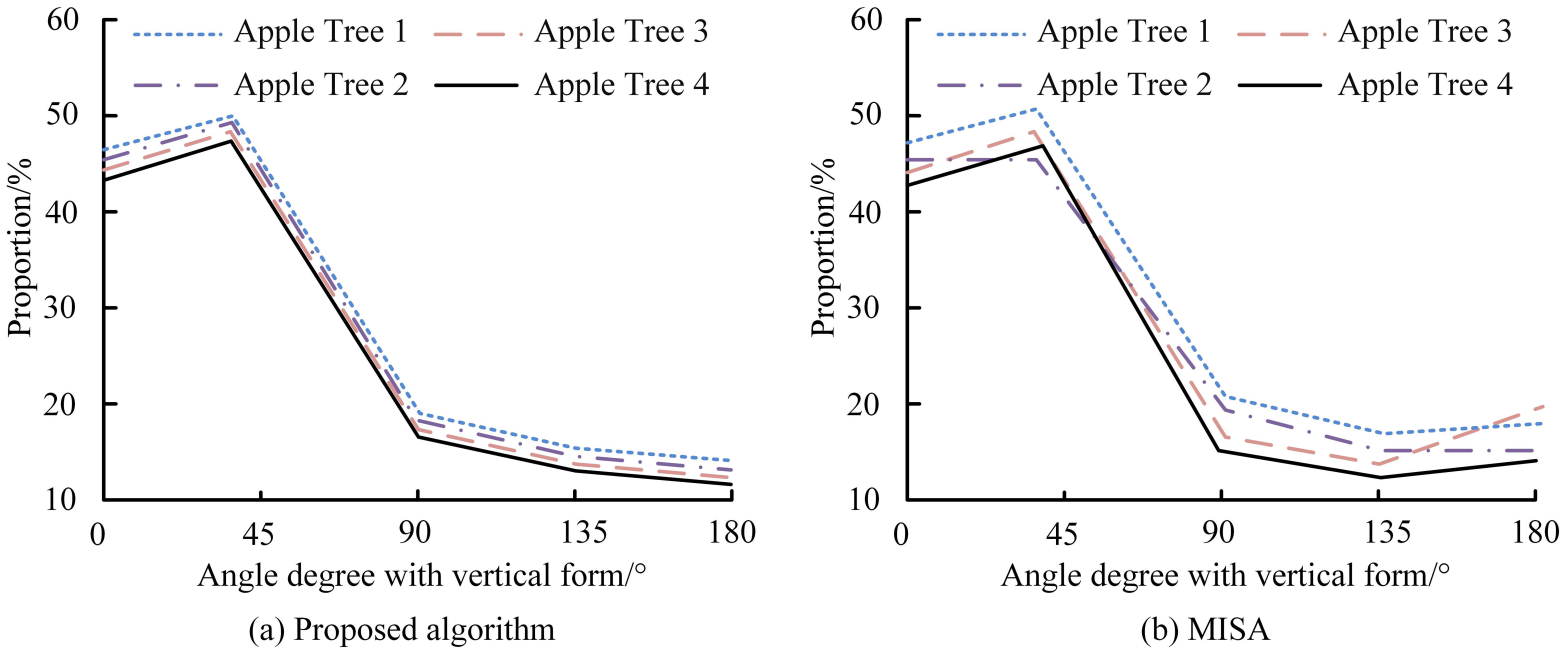
Algorithm pipeline showing preprocessing, multi-feature extraction, feature weighting, fusion, and 3D localization outputs, with results illustrated in **(a)** the proposed algorithm and **(b)** the MISA method.

**Figure 4** illustrates the multi-feature fusion approach for apple image analysis, which involves analyzing multiple pose features from apples and extracting essential features after bias removal to enhance centroid recognition and localization. We calculated the center of mass using the weighted average of pixel coordinates within the region, as described in **Supplementary Equation 5**. Internal and external camera parameters were calibrated using **Supplementary Equation 6**.

The block-matching algorithm extracted parallax values to solve positional discrepancies between left and right camera images (**Supplementary Equation 7**). Depth information was then calculated using parallax values and triangulation principles (**Supplementary Equation 8**). Real-world coordinates were derived by mapping the center of mass and depth information to the camera’s coordinate system (**Supplementary Equation 9**).

The problem of environmental occlusion was solved by applying morphological techniques combined with depth interpolation methods (**Supplementary Equation 10**). Localization accuracy was further enhanced by adjusting camera parameters and refining feature fusion weights based on localization error (Equation 5).

Three-dimensional localization accuracy was tested by taking depth measurements at six points on apple corners at distances ranging from 800 mm to 1100 mm. The difference between real and calculated depth values was assessed, while morphological and depth interpolation techniques minimized errors (**Supplementary Equation 10**).

(5) $$E=\sqrt{{\left(X_{\text{real}}-X_{\text{calc}}\right)}^{2}+{\left(Y_{\text{real}}-Y_{\text{calc}}\right)}^{2}+{\left(Z_{\text{real}}-Z_{\text{calc}}\right)}^{2}}$$

Where *E* represents positioning error and (*X*_real_, *Y*_real_, *Z*_real_) are the actual coordinates and (*X*_calc_, *Y*_calc_, *Z*_calc_) are the calculated coordinates.

### 2.3 Benchmark comparisons and performance evaluation

Benchmarking the proposed model against several state-of-the-art methods allowed for a comprehensive performance evaluation. The selected benchmarks include widely recognized and validated techniques in fruit detection and segmentation research. Faster Region-Based Convolutional Neural Network (Faster R-CNN), You Only Look Once version 7 (YOLOv7), and Masked Region-Based Convolutional Neural Network (Mask R-CNN) are leading deep learning models known for their high detection accuracy. Density-Based Spatial Clustering of Applications with Noise (DBSCAN), Mean-Shift Image Segmentation Algorithm (MISA), and Superpixel Segmentation Algorithm (SSA) are commonly used clustering and segmentation methods designed to handle spatial variation and noise. These methods were chosen to ensure a balanced comparison between deep learning and clustering-based approaches.

The segmentation performance was compared using Mean Coordinate Deviation (MCD) and Correct Recognition Rate (CRR) as evaluation metrics. For object detection and spatial localization, the proposed model was evaluated against YOLOv7, Single Shot MultiBox Detector (SSD), Fully Convolutional Networks (FCN), and Mask R-CNN under four real-world conditions: complex illumination, fruit occlusion, dynamic oscillation, and dense target distribution. Performance was measured using Recognition Accuracy (RA), mean Average Precision (mAP), and Frames Per Second (FPS). Additionally, the model’s stability was assessed across different camera angles (0°, 15°, 30°, and 45°) by comparing it with the Hierarchical Clustering Algorithm (HCA) and Region Growing Segmentation Algorithm (RGSA) using the standard deviation of recognition accuracy.

The proposed model was comprehensively evaluated using RA for detection accuracy, MCD for spatial precision, CRR for segmentation accuracy, F1-score for detection reliability, mAP for overall detection performance, FPS for real-time efficiency, and standard deviation for stability under varying conditions. These metrics collectively demonstrate the model’s accuracy, robustness, and practical efficiency for automated apple detection.

## 3 Results

**Figure 5:**
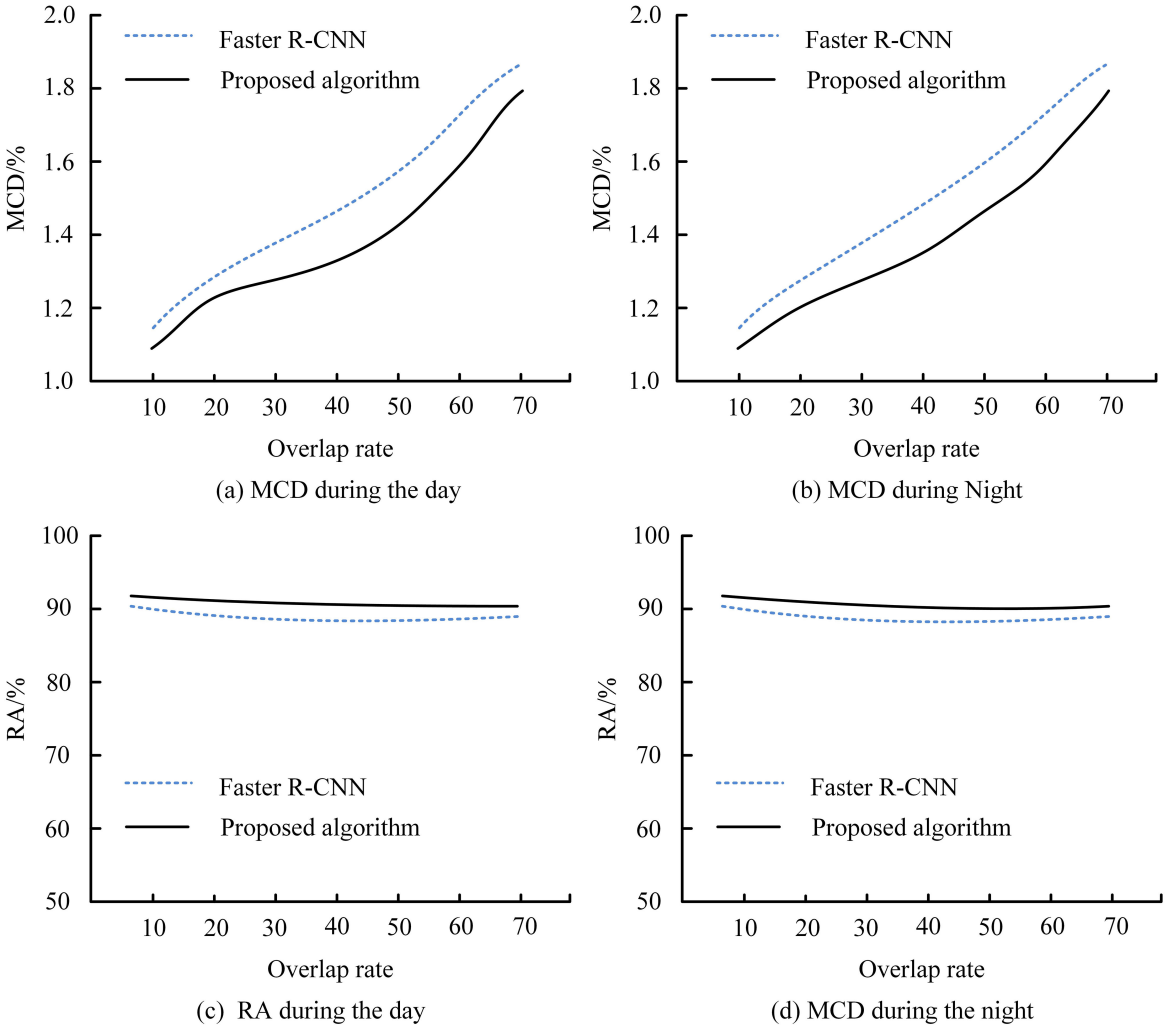
Detection accuracy (RA) and mean coordinate deviation (MCD) of the proposed clustering algorithm and Faster R-CNN under different overlap rates, illustrated for **(a)** MCD during the day, **(b)** MCD during the night, **(c)** RA during the day, and **(d)** RA during the night.

**Figure 6:**
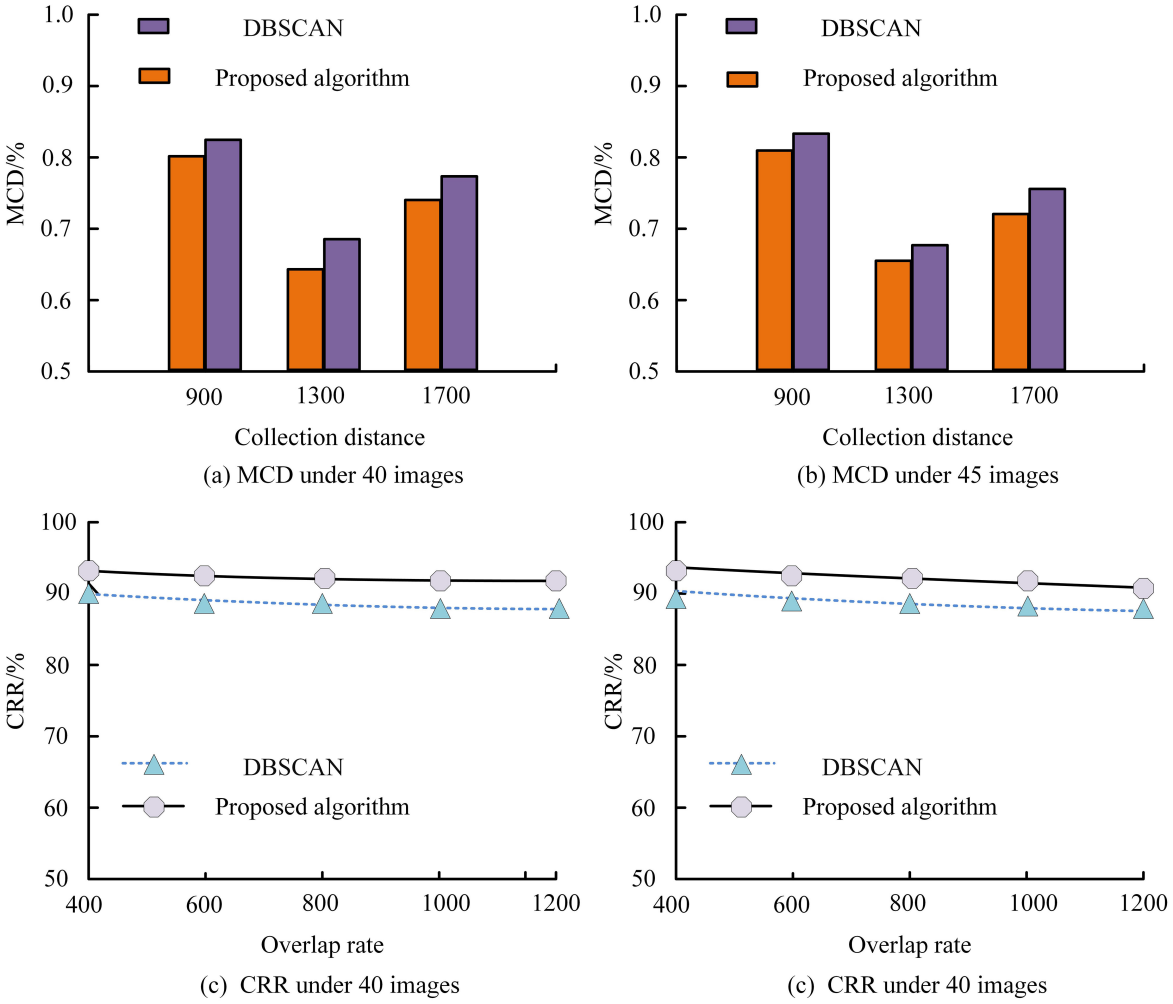
Comparison between the proposed algorithm and DBSCAN across different collection distances (900–1700 mm), shown for **(a)** MCD under 40 images, **(b)** MCD under 45 images, **(c)** CRR under 40 images, and **(d)** CRR under 45 images.

**Figure 7:**
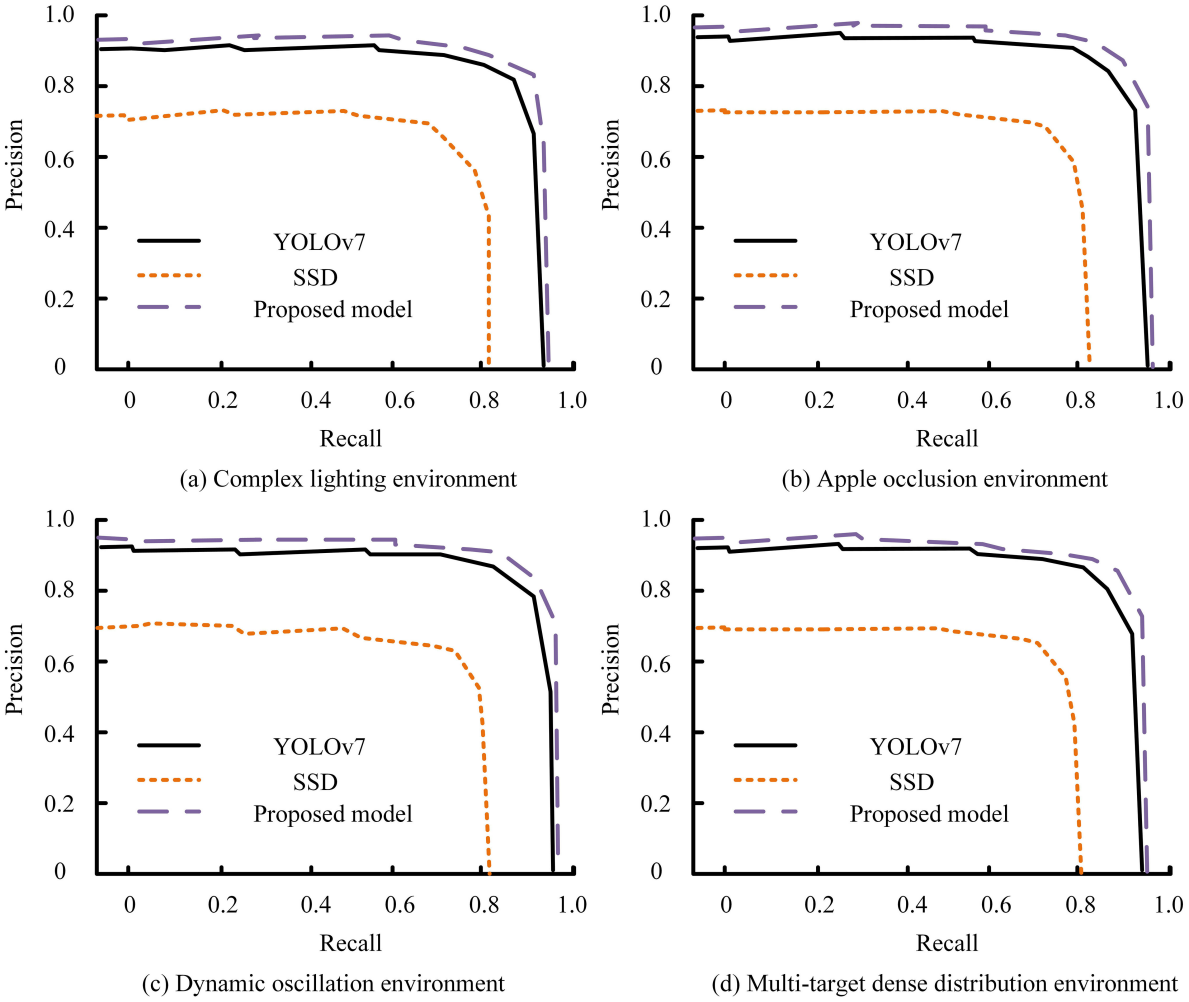
Precision–Recall comparison of YOLOv7, SSD, and the proposed model under different field conditions, including **(a)** complex lighting, **(b)** apple occlusion, **(c)** dynamic oscillation, and **(d)** multi-target dense distribution environments.

The proposed clustering-based segmentation and 3D localization algorithm demonstrated consistent superiority in detection precision and spatial localization under diverse orchard conditions. **Figure 5** illustrates the variation in RA and MCD under different lighting and occlusion levels. The proposed method maintained an average accuracy above 91%, while Faster R-CNN exhibited a pronounced decline when fruit overlaps exceeded 40%. In contrast, our algorithm achieved lower MCD values (≤ 0.3%), indicating more stable spatial localization across both daytime and nighttime datasets. (**Figure 5**). Moreover, the consistently reduced MCD values throughout all collecting distances indicate better localization accuracy of the proposed algorithm (**Figures 6A, B**). **Figures 6C and D** demonstrate that the proposed method consistently maintains a CRR above 90%, outperforming DBSCAN across varying overlap rates. The depth estimation accuracy of the stereo vision system was evaluated by comparing it with YOLOv7 and SSD across four different scenarios: complex lighting conditions, fruit occlusion, dynamic oscillation conditions, and dense target distributions. Across all four tested scenarios, the suggested model showed better recall and precision than YOLOv7 and SSD (**Figure 7**).

**Figure 8:**
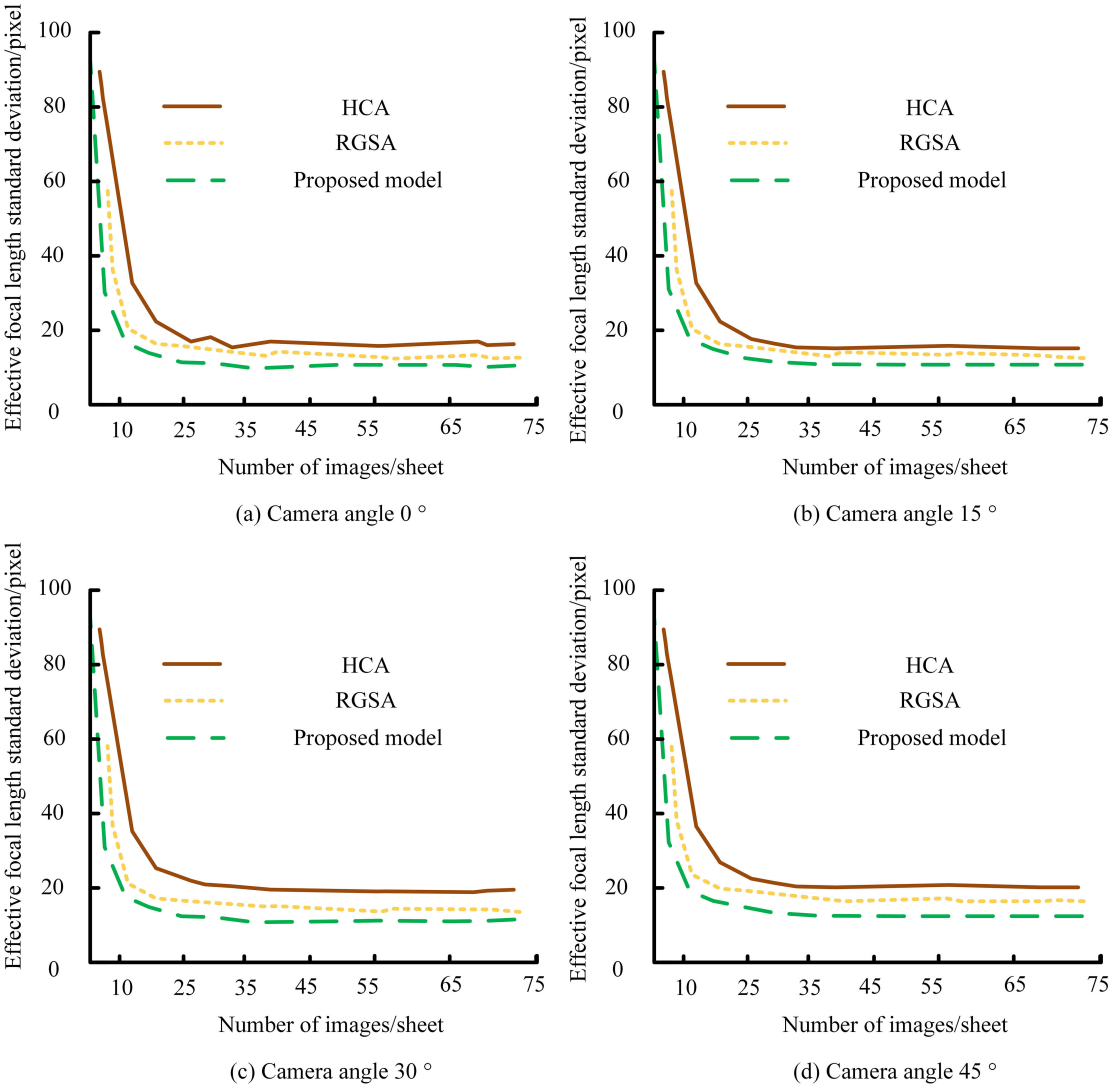
Effective focal-length standard deviation of the stereo vision system under different numbers of images per sheet, evaluated for **(a)** camera angle 0°, **(b)** camera angle 15°, **(c)** camera angle 30°, and **(d)** camera angle 45°, comparing the proposed model with HCA and RGSA.

**Figure 9:**
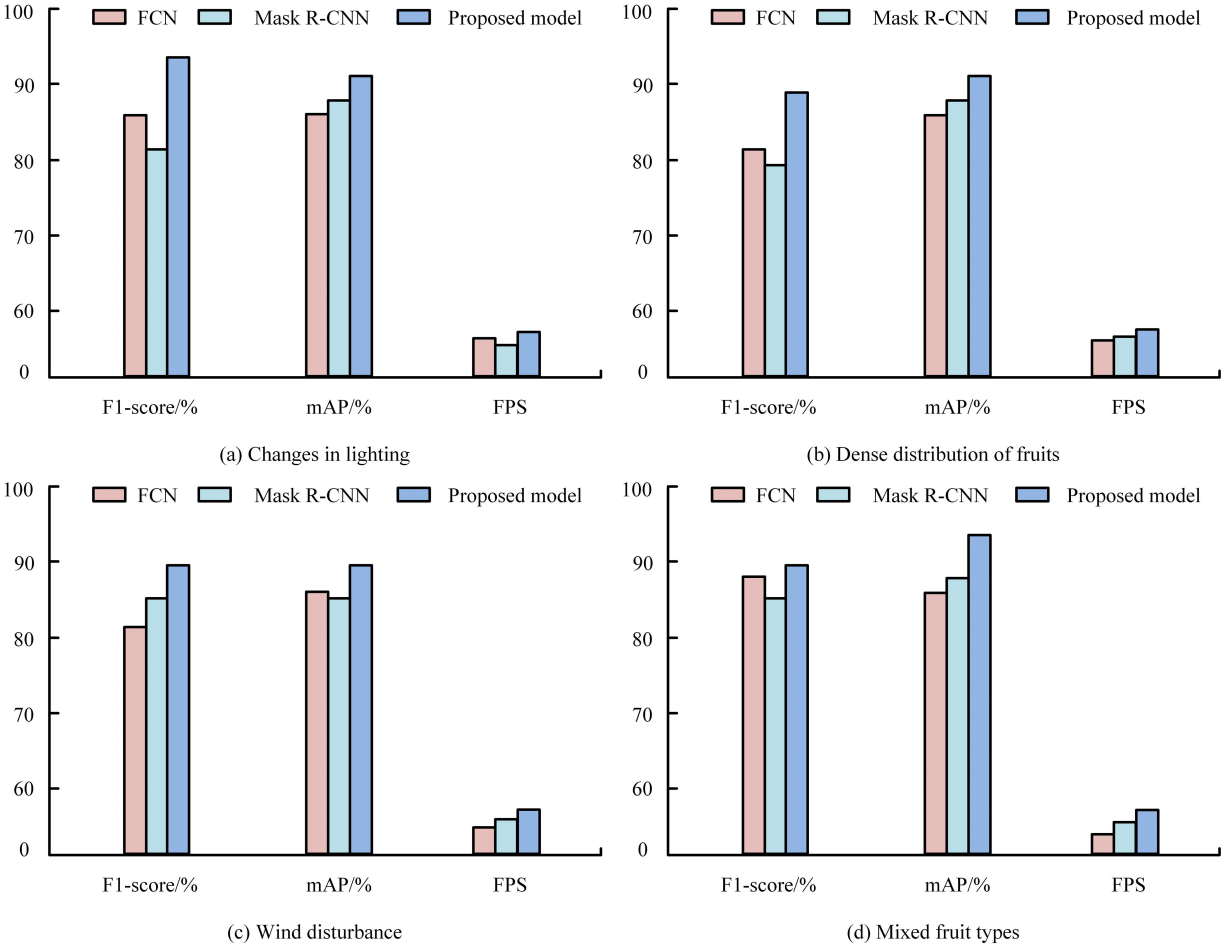
Comparison of apple detection performance among FCN, Mask R-CNN, and the proposed model under different field conditions, including **(a)** changes in lighting, **(b)** dense fruit distribution, **(c)** wind disturbance, and **(d)** mixed fruit types, evaluated using F1-score, mAP, and FPS.

Depth estimation accuracy was further validated, achieving a maximum localization error of 0.97% across 800–1100 mm collection distances (**Figure 8**). Errors ranged from 0.4–0.65% at 800 mm and 0.4–0.5% at 1000 mm, with only slight increases to 0.73–0.79% at 1100 mm. All deviations remained below 1%, confirming high-precision depth estimation suitable for robotic harvesting applications. As shown in **Figures 9A, B**, the proposed algorithm outperformed MISA in detecting apple orientations on four trees at 0°, 45°, 90°, and 180°. It achieved the highest detection rate (> 40%) at 45°, while no apples were detected at 180°, where MISA showed greater variation and overlap, indicating reduced stability. Results for multiple algorithms at the 45° orientation are summarized in **Table 1**. The proposed method achieved the highest recognition accuracy (93%), correctly identifying 39 apples, followed by the CNN model (88%). The template-matching (TM) approach had the lowest accuracy (70%, 28 apples correctly identified).

**Table 1:** Comparative performance of various algorithms in apple posture recognition.

Identification methods	Recognition accuracy (%)	Apples correctly identified (count)
Template Matching (TM)	70	28
Support Vector Machine	75	30
Bayesian Classification	78	31
Convolutional Neural Network (CNN)	88	36
Decision Tree	84	34
Proposed Method	93	39

**Figure 10:**
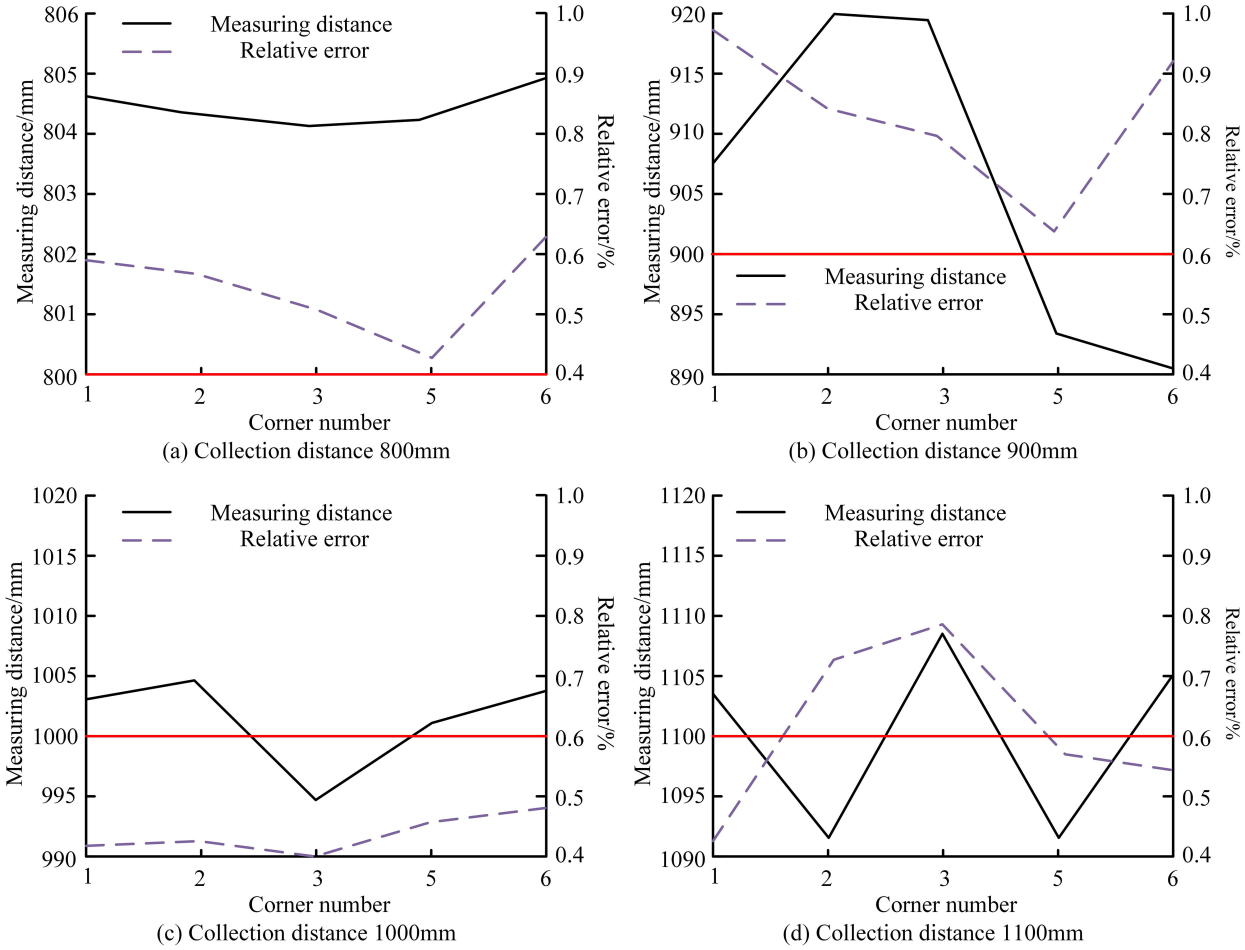
Measuring distance and relative error of the proposed stereo-vision depth estimation system across different collection distances, evaluated at **(a)** 800 mm, **(b)** 900 mm, **(c)** 1000 mm, and **(d)** 1100 mm, based on measurements from six corner points in the calibration board.

**Figure 11:**
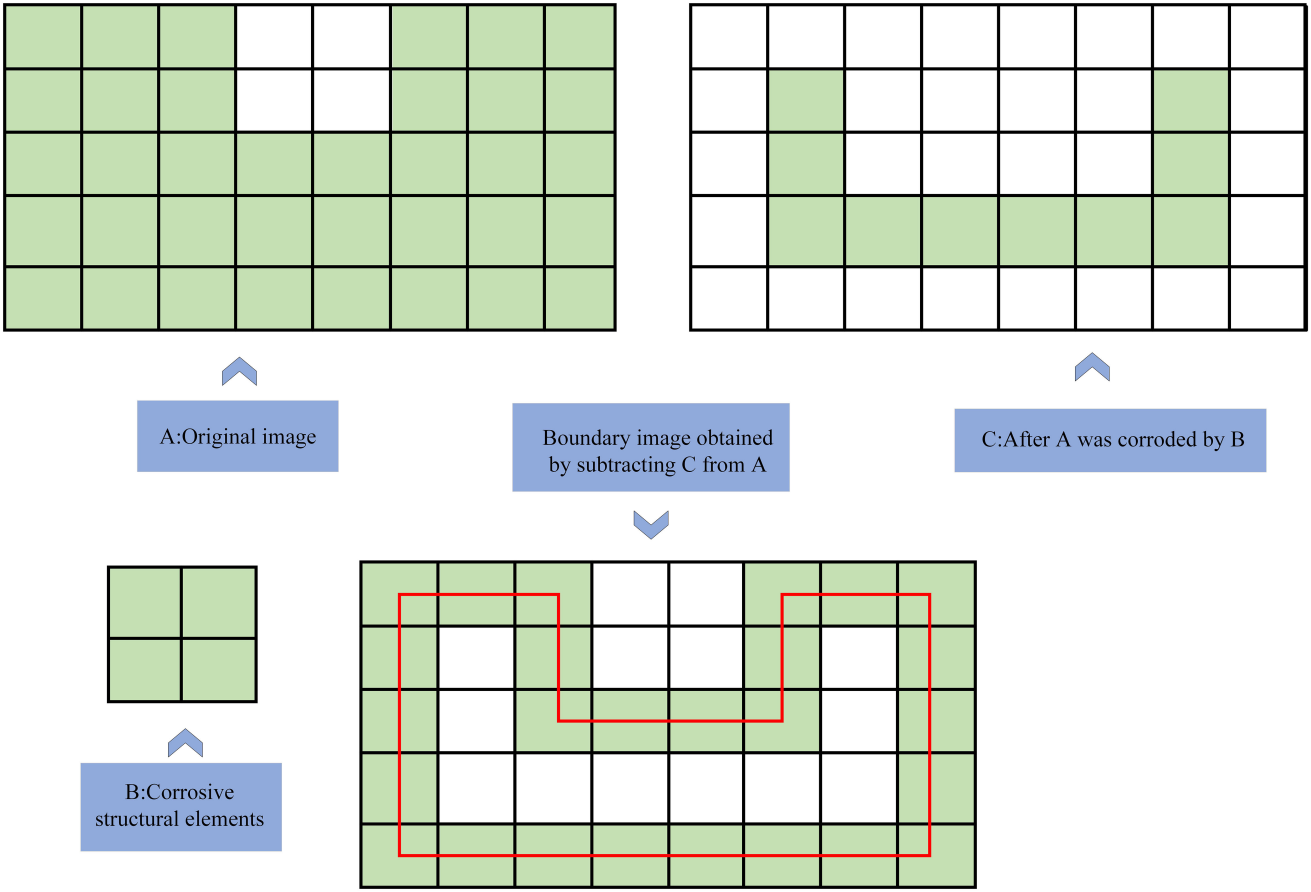
Model stability across camera angles (0°–45°) compared with HCA and RGSA.

In four real-world orchard scenarios, the proposed model was compared with FCN and Mask R-CNN (**Figure 10**). It consistently outperformed both, achieving an F1-score of 92% under varied illumination (**Figure 10A**) and an mAP of 91% for densely clustered fruits (**Figure 10B**). Under wind disturbance (**Figure 10C**), it maintained the highest frame rate per second (FPS), demonstrating strong real-time efficiency. Across multi-fruit orchard conditions (**Figure 10D**), the model again achieved the highest mAP, confirming its robustness and adaptability. **Figure 11** shows that the proposed model maintained the lowest standard deviation across all camera angles (0°–45°), stabilizing after about 25 images. Even at 45°, where deviation slightly increased for all models, it remained the most stable, confirming reliable performance under varying camera orientations.

The proposed clustering-based stereo-vision approach achieved > 91% detection accuracy, < 1% localization error, and stable performance under varying lighting and camera angles, all with a modest dataset. These results demonstrate its suitability for real-time, low-cost robotic harvesting, offering reliable detection and positioning without extensive training or high computational demand—an effective solution for autonomous orchard operations in precision agriculture.

## 4 Discussion

Accurate segmentation is crucial for precise apple detection in challenging orchard environments (Kang and Chen, 2020). The improved MCD and RA values indicate that multi-feature fusion with adaptive K-means clustering increases robustness to lighting changes and occlusion. Deep-learning models such as Faster R-CNN often lose accuracy under these conditions (Bargoti and Underwood, 2017; Fu et al., 2020). In contrast, the proposed unsupervised approach remains stable with fewer samples. Compared with DBSCAN, it achieved higher stability and accuracy across distances and image counts (Limwattanapibool and Arch‐int, 2017; Hartigan and Wong, 1979). These results confirm strong generalization and real-time potential for orchard use.

The success of robotic apple picking depends heavily on precise 3D localization. Our results are consistent with earlier research, where YOLO-based algorithms struggle to make real-time changes in challenging agricultural settings (Jiang et al., 2022b). This is consistent with other studies where YOLO-based models struggle in complex environments (Bresilla et al., 2019; Parvathi and Selvi, 2021). Consistent with previous studies, YOLOv7 demonstrated better accuracy and recognition speed than SSD (Wang and Chen, 2024). In contrast, a previous study showed that YOLOv7 achieved exceptional detection rates of *Camellia oleifera* fruit in orchards with 95.74% mAP, 93.67% F1 score, 94.21% precision, 93.13% recall and a detection time of 0.025 seconds (Wu et al., 2022). Recent research on brinjal detection using deep learning models has demonstrated the effectiveness of a lightweight YOLO architecture and edge-based computing frameworks for real-time harvesting applications (Nahiduzzaman et al., 2025; Tamilarasi et al., 2025). These approaches, while achieving high precision and recall, still depend on large, annotated datasets and relatively intensive computational resources. In contrast, our clustering-based multi-feature method achieves stable performance with fewer training samples and reduced hardware requirements, underscoring its suitability for orchard conditions. Our results are consistent with previous studies, indicating that while SSD performs well in controlled environments, it may struggle in more complex scenarios than YOLOv7. For example, Xu et al. reported lower SSD performance in typical agricultural environments where occlusions and cluttered backgrounds are very common (Xu et al., 2024). In contrast, Deng et al (Deng et al., 2024). found that YOLOv7, when enhanced with attention mechanisms, consistently outperformed SSD in citrus detection under different orchard conditions. Apple posture detection is critical in establishing the best picking strategies (Liu et al., 2024). The observed stable detection suggests that our method effectively addresses occlusion and angle-related distortions, a common challenge in fruit detection (Safari et al., 2024).

The proposed method showed stable performance relative to MISA and achieved higher accuracy than CNN, TM, and other traditional classifiers, reflecting improved feature extraction and classification capability. Similar challenges in illumination and feature consistency were also noted by (Sun et al., 2021). Consistent results under varying field conditions confirm that the model can maintain real-time reliability in orchard operations. Previous studies using FCN reported fruit-counting accuracies of 0.91–0.95 and yield accuracies up to 0.98 (Häni et al., 2020), while Faster R-CNN achieved an F1-score of 0.89 and 91% mAP. In contrast, our model achieved higher mAP, F1-score, and frame rate, demonstrating superior detection in dense, multi-fruit environments. Real-world comparison with FCN and Mask R-CNN confirmed the proposed model*’*s superior accuracy and processing efficiency for dense, multi-fruit environments (Wan and Goudos, 2020; He et al., 2017). Compared to previous studies, Mask R-CNN performed poorly in our study, where the precision rate reached 97.31% and the recall rate reached 95.70% (Jia et al., 2020). These outcomes highlight its stability and real-time applicability under orchard conditions. Unlike deep-learning models that rely on large annotated datasets, the algorithm maintained strong performance with limited training images, reflecting better adaptability and lower data dependence (Koirala et al., 2019). Bargoti and Underwood found that 729 training images were necessary to stabilize AP for apple detection, but almond and mango models needed more data (Bargoti and Underwood, 2017). This study also demonstrated that data augmentation enabled better apple detection using only 100 images compared to 300 images without augmentation. Similarly, 93% of apples were accurately detected in 50 images despite uneven lighting conditions in a previous study (Xu and Lv, 2018). Compared to deep learning models like Faster R-CNN and YOLOv7, the proposed method requires less computational power and no extensive training, making it suitable for real-time applications on standard hardware. While sequential processing may limit scalability in large-scale deployments, this can be optimized with parallel computing. The pipeline*’*s reliance on generalizable features such as colour, texture, and morphology also makes it adaptable to other fruits or crops with minor adjustments. However, large-scale field validation and integration with robotic harvesting systems are still required to confirm performance under real operating conditions, which will be addressed in future development.

In conclusion, this study presents a clustering-based stereo vision algorithm that combines K-means segmentation and multi-feature fusion for accurate apple detection and 3D localization in orchard environments. The method offers high accuracy, strong generalization, and real-time feasibility with minimal training data and computational demand—key advantages over deep-learning approaches. While sequential processing and limited field scale remain constraints, these can be addressed through parallel computing and large-scale robotic trials. Future work should focus on optimizing real-time performance and extending the framework to other fruit crops and intelligent harvesting systems.

## Data Availability

The original contributions presented in the study are included in the article/**Supplementary Material**. Further inquiries can be directed to the corresponding author.

## References

[B1] Ashtiani S.-H. M. Javanmardi S. Jahanbanifard M. Martynenko A. Verbeek F. J. (2021). Detection of mulberry ripeness stages using deep learning models. IEEE Access 9, 100380–100394. doi: 10.1109/ACCESS.2021.3096550

[B2] Bargoti S. Underwood J. (2017). “ Deep fruit detection in orchards,” in IEEE International Conference on Robotics and Automation (ICRA): IEEE). New York (USA): Institute of Electrical and Electronics Engineers (IEEE). 3626–3633.

[B3] Biffi L. J. Mitishita E. Liesenberg V. Santos A. Goncalves D. N. Estrabis N. V. . (2020). ATSS deep learning-based approach to detect apple fruits. Remote Sens. 13, 54. doi: 10.3390/rs13010054

[B4] Bresilla K. Perulli G. D. Boini A. Morandi B. Corelli Grappadelli L. Manfrini L. (2019). Single-shot convolution neural networks for real-time fruit detection within the tree. Front. Plant Sci. 10. doi: 10.3389/fpls.2019.00611, PMID: 31178875 PMC6537632

[B5] Deng F. Chen J. Fu L. Zhong J. Qiaoi W. Luo J. . (2024). Real-time citrus variety detection in orchards based on complex scenarios of improved YOLOv7. Front. Plant Sci. 15. doi: 10.3389/fpls.2024.1381694, PMID: 39011299 PMC11246913

[B6] Fu L. Majeed Y. Zhang X. Karkee M. Zhang Q. (2020). Faster R–CNN–based apple detection in dense-foliage fruiting-wall trees using RGB and depth features for robotic harvesting. Biosyst. Eng. 197, 245–256. doi: 10.1016/j.biosystemseng.2020.07.007

[B7] Gao G. Bai Q. Zhang C. Zhang L. Yao L. (2023a). Dualistic cascade convolutional neural network dedicated to fully PolSAR image ship detection. ISPRS J. Photogrammetry Remote Sens. 202, 663–681. doi: 10.1016/j.isprsjprs.2023.07.006

[B8] Gao G. Wang M. Zhou P. Yao L. Zhang X. Li H. . (2024). A multi-branch embedding network with bi-classifier for few-shot ship classification of SAR images. IEEE Trans. Geosci. Remote Sens. 63, 5201515. doi: 10.1109/TGRS.2024.3500034

[B9] Gao G. Zhang C. Zhang L. Duan D. (2023b). Scattering characteristic-aware fully polarized SAR ship detection network based on a four-component decomposition model. IEEE Trans. Geosci. Remote Sens. 61, 1–22. doi: 10.1109/TGRS.2023.3336300

[B10] Gonzales R. C. Woods R. E. (2018). Digital image processing 4th edition. (New York: Pearson).

[B11] Guan Y. Zhang X. Gao G. Cao C. Li Z. Fu S. . (2025). A new indicator for assessing fishing ecological pressure using multi-source data: A case study of the South China Sea. Ecol. Indic. 170, 113096. doi: 10.1016/j.ecolind.2025.113096

[B12] Häni N. Roy P. Isler V. (2020). A comparative study of fruit detection and counting methods for yield mapping in apple orchards. J. Field Robotics 37, 263–282. doi: 10.1002/rob.21902

[B13] Hartigan J. A. Wong M. A. (1979). Algorithm AS 136: A k-means clustering algorithm. J. R. Stat. Society Ser. C (Applied Statistics) 28, 100–108. doi: 10.2307/2346830

[B14] Hartley R. Zisserman A. (2003). Multiple view geometry in computer vision. (Cambridge, UK: Cambridge University Press).

[B15] He K. Gkioxari G. Dollár P. Girshick R. (2017). “ Mask R-CNN,” in Proceedings of the IEEE international conference on computer vision: IEE). New York, USA: Institute of Electrical and Electronics Engineers (IEEE). 2961–2969.

[B16] Ikotun A. M. Ezugwu A. E. Abualigah L. Abuhaija B. Heming J. (2023). K-means clustering algorithms: A comprehensive review, variants analysis, and advances in the era of big data. Inf. Sci. 622, 178–210. doi: 10.1016/j.ins.2022.11.139

[B17] Jamel A. Akay B. (2019). A survey and systematic categorization of parallel K-Means and Fuzzy-C-Means algorithms. Comput. Syst. Sci. Eng. 34, 259–281. doi: 10.32604/csse.2019.34.259

[B18] Javanmardi S. Ashtiani S.-H. M. (2025). AI-driven deep learning framework for shelf life prediction of edible mushrooms. Postharvest Biol. Technol. 222, 113396. doi: 10.1016/j.postharvbio.2025.113396

[B19] Jia W. Tian Y. Luo R. Zhang Z. Lian J. Zheng Y. (2020). Detection and segmentation of overlapped fruits based on optimized mask R-CNN application in apple harvesting robot. Comput. Electron. Agric. 172, 105380. doi: 10.1016/j.compag.2020.105380

[B20] Jiang P. Ergu D. Liu F. Cai Y. Ma B. (2022b). A Review of Yolo algorithm developments. Proc. Comput. Sci. 199, 1066–1073. doi: 10.1016/j.procs.2022.01.135

[B21] Jiang J. Johansen K. Stanschewski C. S. Wellman G. Mousa M. A. Fiene G. M. . (2022a). Phenotyping a diversity panel of quinoa using UAV-retrieved leaf area index, SPAD-based chlorophyll and a random forest approach. Precis. Agric. 23, 961–983. doi: 10.1007/s11119-021-09870-3

[B22] Johansen K. Morton M. J. Malbeteau Y. Aragon B. Al-Mashharawi S. Ziliani M. G. . (2020). Predicting biomass and yield in a tomato phenotyping experiment using UAV imagery and random forest. Front. Artif. Intell. 3. doi: 10.3389/frai.2020.00028, PMID: 33733147 PMC7861253

[B23] Johansen K. Morton M. Malbeteau Y. Aragon Solorio B. J. L. Almashharawi S. Ziliani M. . (2019). Predicting biomass and yield at harvest of salt-stressed tomato plants using UAV imagery. Int. Arch. Photogrammetry Remote Sens. Spatial Inf. Sci. - ISPRS Arch. XLII-2/W13, 407–411. doi: 10.5194/isprs-archives-XLII-2-W13-407-2019

[B24] Kang H. Chen C. (2020). Fruit detection, segmentation and 3D visualisation of environments in apple orchards. Comput. Electron. Agric. 171, 105302. doi: 10.1016/j.compag.2020.105302

[B25] Koirala A. Walsh K. B. Wang Z. Mccarthy C. (2019). Deep learning–method overview and review of use for fruit detection and yield estimation. Comput. Electron. Agric. 162, 219–234. doi: 10.1016/j.compag.2019.04.017

[B26] Likas A. Vlassis N. Verbeek J. J. (2003). The global k-means clustering algorithm. Pattern Recognition 36, 451–461. doi: 10.1016/S0031-3203(02)00060-2

[B27] Limwattanapibool O. Arch-Int S. (2017). Determination of the appropriate parameters for K-means clustering using selection of region clusters based on density DBSCAN (SRCD-DBSCAN). Expert Syst. 34, e12204. doi: 10.1111/exsy.12204

[B28] Liu S. Xue J. Zhang T. Lv P. Qin H. Zhao T. (2024). Research progress and prospect of key technologies of fruit target recognition for robotic fruit picking. Front. Plant Sci. 15. doi: 10.3389/fpls.2024.1423338, PMID: 39711588 PMC11659763

[B29] Mhamed M. Zhang Z. Yu J. Li Y. Zhang M. (2024). Advances in apple’s automated orchard equipment: A comprehensive research. Comput. Electron. Agric. 221, 108926. doi: 10.1016/j.compag.2024.108926

[B30] Na S. Xumin L. Yong G. (2010). “ Research on k-means clustering algorithm: An improved k-means clustering algorithm,” in Third International Symposium on Intelligent Information Technology and Security Informatics, IITSI: IEEE). New York, USA: Institute of Electrical and Electronics Engineers (IEEE). 63–67.

[B31] Nahiduzzaman M. Sarmun R. Khandakar A. Faisal M. Islam M. S. Alam M. K. . (2025). Deep learning-based real-time detection and classification of tomato ripeness stages using YOLOv8 on raspberry Pi. Eng. Res. Express 7, 015219. doi: 10.1088/2631-8695/ada720

[B32] Onishi Y. Yoshida T. Kurita H. Fukao T. Arihara H. Iwai A. (2019). An automated fruit harvesting robot by using deep learning. ROBOMECH J. 6, 1–8. doi: 10.1186/s40648-019-0141-2

[B33] Parvathi S. Selvi S. T. (2021). Detection of maturity stages of coconuts in complex background using Faster R-CNN model. Biosyst. Eng. 202, 119–132. doi: 10.1016/j.biosystemseng.2020.12.002

[B34] Qu W. Shang W. Shao Y. Wang D. Yu X. Song H. (2015). Segmentation of foreground apple targets by fusing visual attention mechanism and growth rules of seed points. Spanish J. Agric. Res. 13, e0214. doi: 10.5424/sjar/2015133-7047

[B35] Ramadhani S. Azzahra D. Tomi Z. (2022). Comparison of K-Means and K-Medoids algorithms in text mining based on Davies Bouldin Index testing for classification of student’s thesis. Jurnal Teknologi Informasi dan Komunikasi 13, 24–33. doi: 10.31849/digitalzone.v13i1.9292

[B36] Safari Y. Nakatumba-Nabende J. Nakasi R. Nakibuule R. (2024). A Review on automated detection and assessment of fruit damage using machine learning. IEEE Access 12, 1–12. doi: 10.1109/ACCESS.2024.3362230

[B37] Sarbaini S. Saputri W. Muttakin F. (2022). Cluster analysis menggunakan algoritma fuzzy K-means Untuk Tingkat Pengangguran Di Provinsi Riau. Jurnal Teknologi Dan Manajemen Industri Terapan 1, 78–84. doi: 10.55826/tmit.v1iII.30

[B38] Shen B. Liu T. Gao G. Chen H. Yang J. (2024). A low-cost polarimetric radar system based on mechanical rotation and its signal processing. IEEE Trans. Aerospace Electronic Syst. 61, 4744–4765. doi: 10.1109/TAES.2024.3507776

[B39] Shi X. Wang S. Zhang B. Ding X. Qi P. Qu H. . (2025). Advances in object detection and localization techniques for fruit harvesting robots. Agronomy 15, 145. doi: 10.3390/agronomy15010145

[B40] Song H. Zhang C. Pan J. Yin X. Zhuang Y. (2013). Segmentation and reconstruction of overlappedapple images based on convex hull. Trans. Chin. Soc. Agric. Eng. 29, 163–168. doi: 10.3969/j.issn.1002-6819.2012.22.025

[B41] Sun S. Li C. Chee P. W. Paterson A. H. Meng C. Zhang J. . (2021). High resolution 3D terrestrial LiDAR for cotton plant main stalk and node detection. Comput. Electron. Agric. 187, 106276. doi: 10.1016/j.compag.2021.106276

[B42] Tamilarasi T. Muthulakshmi P. Ashtiani S.-H. M. (2025). Smart edge computing framework for real-time brinjal harvest decision optimization. AgriEngineering 7, 196. doi: 10.3390/agriengineering7060196

[B43] Tianjing Y. Mhamed M. (2024). Developments in automated harvesting equipment for the apple in the orchard. Smart Agric. Technol. 9, 100491. doi: 10.1016/j.atech.2024.100491

[B44] Tu J. Liu C. Li Y. Zhou J. Yuan J. (2010). Apple recognition method based on illumination invariant graph. Trans. Chin. Soc. Agric. Eng. 26, 26–31. doi: 10.3969/j.issn.1002-6819.2014.24.020

[B45] Wan S. Goudos S. (2020). Faster R-CNN for multi-class fruit detection using a robotic vision system. Comput. Networks 168, 107036. doi: 10.1016/j.comnet.2019.107036

[B46] Wang H. Chen X. (2024). “ Object detection of classroom students based on improved YOLOv7,” in Third International Symposium on Computer Applications and Information Systems (ISCAIS 2024): SPIE). Bellingham, Washington, USA: SPIE - The International Society for Optics and Photonics. 484–489.

[B47] Wang C. Liu S. Wang Y. Xiong J. Zhang Z. Zhao B. . (2022). Application of convolutional neural network-based detection methods in fresh fruit production: a comprehensive review. Front. Plant Sci. 13. doi: 10.3389/fpls.2022.868745, PMID: 35651761 PMC9149381

[B48] Wang Dandan W. D. Xu Yue X. Y. Song Huaibo S. H. He Dongjian H. D. Zhang Haihui Z. H. (2015). Fusion of K-means and Ncut algorithm to realize segmentation and reconstruction of two overlapped apples without blocking by branches and leaves. Trans. Chin. Soc. Agric. Eng. 31, 227–234. doi: 10.11975/j.issn.1002-6819.2015.10.030

[B49] Wu D. Jiang S. Zhao E. Liu Y. Zhu H. Wang W. . (2022). Detection of *Camellia oleifera* fruit in complex scenes by using YOLOv7 and data augmentation. Appl. Sci. 12, 11318. doi: 10.3390/app122211318

[B50] Xiao F. Wang H. Xu Y. Zhang R. (2023). Fruit detection and recognition based on deep learning for automatic harvesting: An overview and review. Agronomy 13, 1625. doi: 10.3390/agronomy13061625

[B51] Xu L. Lv J. (2018). Recognition method for apple fruit based on SUSAN and PCNN. Multimedia Tools Appl. 77, 7205–7219. doi: 10.1007/s11042-017-4629-6

[B52] Xu D. Ren R. Zhao H. Zhang S. (2024). Intelligent detection of muskmelon ripeness in greenhouse environment based on YOLO-RFEW. Agronomy 14, 1091. doi: 10.3390/agronomy14061091

[B53] Yang H. Lauren C. Nebojsa D. Erik W. Predrag B. (2012). “ Performance analysis of EM-MPM and K-means clustering in 3D ultrasound breast image segmentation,” in IEEE International Conference on Electro/Information Technology, Indianapolis, IN, USA ( IEE). New York (USA): Institute of Electrical and Electronics Engineers (IEEE).

[B54] Zhang X. Gao G. Chen S.-W. (2024). Polarimetric autocorrelation matrix: A new tool for joint characterizing of target polarization and Doppler scattering mechanism. IEEE Trans. Geosci. Remote Sens. 62, 65–75. doi: 10.1109/TGRS.2024.3398632

[B55] Zhang Z. Igathinathane C. Li J. Cen H. Lu Y. Flores P. (2020). Technology progress in mechanical harvest of fresh market apples. Comput. Electron. Agric. 175, 105606. doi: 10.1016/j.compag.2020.105606

[B56] Zubair M. Iqbal M. A. Shil A. Chowdhury M. Moni M. A. Sarker I. H. (2024). An improved K-means clustering algorithm towards an efficient data-driven modeling. Ann. Data Sci. 11, 1525–1544. doi: 10.1007/s40745-022-00428-2, PMID: 40479183 PMC9243813

