# Supplementary material for: Cluster segmentation and stereo vision-based apple localization algorithm for robotic harvesting
Source: Front Plant Sci. 2025 Nov 27;16:1598414. doi: 10.3389/fpls.2025.1598414 (PMC12695797; doi:10.3389/fpls.2025.1598414)
Supplement: Supplementary file 1 [file Table1.docx]

# Supplementary Equations

## Equation 1

G(i,j) = ( R(i,j) + G(i,j) + B(i,j) ) / 3

Where G(i,j) is the grayscale value, and R(i,j), G(i,j), B(i,j) are the red, green, and blue components at pixel (i,j).

## Equation 2

G(x,y) = (1 / (2 * pi * sigma^2)) * exp( - (x^2 + y^2) / (2 * sigma^2) )

Where G(x,y) is the Gaussian filter kernel, x and y are coordinates, and sigma is the standard deviation.

## Equation 3

H = arccos( ( (R - 0.5*G - 0.5*B) ) / sqrt( (R - G)^2 + (R - B)*(G - B) ) )
S = 1 - ( 3 / (R + G + B) ) * min(R,G,B)
I = (R + G + B) / 3

Where H is hue, S is saturation, and I is intensity in the HSI colour model. R, G, and B are red, green, and blue channel values.

## Equation 4

R = (F ⊕ S) ⊖ S

Where R is the processed image, F is the binary input, S is the structuring element, ⊕ is dilation, and ⊖ is erosion.

## Equation 5

L = { Ri | i = 1,2,...,N }

Where L is the set of connected region labels, Ri is the pixel set of region i, and N is the total number of samples.

## Equation 6

Cx = (1 / N0) * Σ[ f(xi, yi) * xi ]
Cy = (1 / N0) * Σ[ f(xi, yi) * yi ]

Where Cx and Cy are centroid coordinates, N0 is the number of pixels, and f(xi, yi) is the feature value at pixel (xi, yi).

## Equation 7

A = [ fx 0 cx ]
 [ 0 fy cy ]
 [ 0 0 1 ], [R1 | T1]

Where A is the intrinsic matrix, fx, fy are focal lengths, cx, cy are the principal point, and R1, T1 are rotation and translation.

## Equation 8

d(x,y) = L(x,y) - R(x,y)

Where d(x,y) is disparity, and L(x,y), R(x,y) are the grey values from left and right images.

## Equation 9

Z = (f * B) / d

Where Z is depth, f is focal length, B is baseline distance, and d is disparity.

## Equation 10

X = ((Cx - cx) * Z) / fx
Y = ((Cy - cy) * Z) / fy
Z = Z

Where (X,Y,Z) are real-world coordinates, (Cx,Cy) are centroid pixel coordinates, and cx, cy, fx, fy are camera parameters.

## Equation 11

Z' = ( Σ( wi * Zi ) ) / ( Σ wi )

Where Z' is the interpolated depth, Zi are surrounding depth values, and wi are weights inversely proportional to distance.
